# Validation of a prognostic scoring system for locally recurrent nasopharyngeal carcinoma treated by stereotactic radiosurgery

**DOI:** 10.1186/1471-2407-9-131

**Published:** 2009-04-29

**Authors:** Daniel TT Chua, Kwan-Ngai Hung, Victor Lee, Sherry CY Ng, Janice Tsang

**Affiliations:** 1 Department of Clinical Oncology, Queen Mary Hospital, The University of Hong Kong, Hong Kong; 2 Department of Surgery, Queen Mary Hospital, The University of Hong Kong, Hong Kong

## Abstract

**Background:**

Selection of patients with local failure of nasopharyngeal carcinoma (NPC) for appropriate type of salvage treatment can be difficult due to the lack of data on comparative efficacy of different salvage treatments. The purpose of the present study was to validate a previously published prognostic scoring system for local failures of NPC treated by radiosurgery based on reported results in the literature.

**Methods:**

A literature search yielded 3 published reports on the use of radiosurgery as salvage treatment of NPC that contained sufficient clinical information for validation of the scoring system. Prognostic scores of 18 patients from these reports were calculated and actuarial survival rates were estimated and compared to the original cohort used to design the prognostic scoring system. The area under the receiver operating characteristic curve was also determined and compared between the current and original patient groups.

**Results:**

The calculated prognostic scores ranged from 0.32 to 1.21, with 15 patients assigned to the poor prognostic group and 3 to the intermediate prognostic group. The actuarial 3-year survival rates in the intermediate and poor prognostic groups were 67% and 0%, respectively. These results were comparable to the observed 3-year survival rates of 74% and 23% in the intermediate and poor prognostic group in the original reports. The area under the receiver operating characteristic curve for the current patient group was 0.846 which was similar to 0.841 in the original group.

**Conclusion:**

The previously published prognostic scoring system demonstrated good prediction of treatment outcome after radiosurgery in a small group of NPC patients with poor prognosis. Prospective study to validate the scoring system is currently being carried out in our institution.

## Background

Loco-regional control rate of nasopharyngeal carcinoma (NPC) has improved significantly in the past decade due to the advances in imaging, radiotherapy technique, and the use of combined modality treatment. The reported 5-year local control rates of NPC in modern series ranged from 81 to 85%, with control rates exceeding 90% for patients with T1 disease [1,2]. Despite the improved outcome in local control, local recurrence still represents a major cause of mortality and morbidity in advanced stage disease, and management of local failure remains an important and challenging issue in NPC.

Aggressive salvage treatment is generally recommended for local recurrence since long-term control can still be achieved in some patients. When the recurrent tumor is confined to the nasopharynx, both nasopharyngectomy and brachytherapy can be used with a long term control rate of 52–72% [3-5]. Patients with more extensive or bulky disease however often required external beam re-irradiation, but treatment outcome with conventional radiotherapy was unsatisfactory with a poor survival rate and a high incidence of late complication [6,7]. The use of intensity-modulated radiotherapy (IMRT) in retreatment of NPC allows better sparing of normal tissues while delivering high dose to the target, and preliminary reports using IMRT for re-irradiation of NPC showed good short-term control with a relatively low incidence of severe toxicities [8]. Stereotactic radiotherapy has also been employed in the treatment of local failures of NPC, with long term control rates of 53 to 86% [9-12]. Compared to other salvage treatments, stereotactic radiotherapy has the advantage of being applicable to deep-seated tumor such as recurrence in the skull base or intracranium. Stereotactic radiotherapy also allows better sparing of critical structures that are in close vicinity to the target. No direct comparison of different salvage treatment techniques for NPC has ever been reported, and the choice of treatment largely depends on the tumor extent, available expertise, and patient/clinician preference. On the other hand, it is important to identify prognostic factors that may predict good or poor outcome using specific salvage treatment and use them clinically to select patient for appropriate type of salvage treatment. A prognostic scoring system for locally recurrent NPC treated by stereotactic radiosurgery was reported by us previously [13]. In order to validate the scoring system, we collected relevant data from published literature on the use of radiosurgery for local failures of NPC and compared the performance of the prognostic scoring system in this patient group with the original one used to design the scoring system.

## Methods

Prognostic factors of a cohort of 48 patients with local failure of NPC treated by stereotactic radiosurgery in our institution were identified and a prognostic scoring was then designed to predict treatment outcome. All these patients were treated by stereotactic radiosurgery using a modified linear accelerator and a median dose of 12.5 Gy (range: 12 to 18 Gy) was delivered to the target. Briefly, 5 factors were included in the scoring system: age, recurrent/persistent disease, recurrent T (rT) stage, tumor volume, and previous salvage treatment. In staging the recurrent tumor, the same 1997 American Joint Committee on Cancer stage classification system [14] used for staging of primary tumor in newly diagnosed NPC was adopted: T1 if tumor is localized in nasopharynx, T2 if tumor has extend to nasal fossa, parapharyngeal space or oropharynx, T3 if there is evidence of skull base or other para-nasal sinus involvement, and T4 if there is extension into intracranium, orbit, hypopharynx or cranial nerve involvement. The relative risks associated with these factors were represented by the estimated risk coefficient obtained by the Cox proportional hazards regression model and the value ranged from 0.05 to 0.39. A score of 0 was assigned if the adverse factor was not present, or a value equal to the estimated risk coefficient if the factor was present. The relative risk of failure after radiosurgery for each patient (prognostic score) was then estimated by summing the relative risk of individual factors. Patients were then grouped to the following subgroups based on the calculated prognostic score: good prognostic group with a score of 0, intermediate prognostic group with a score > 0 to 0.5, and poor prognostic group with a score > 0.5. 5-year local relapse-free rates in the good, intermediate and poor prognostic groups were 100%, 42.5% and 9.6%, respectively. The corresponding 5-year survival rates were 100%, 51.1% and 0%.

Validation of the scoring system was performed using published data from the literature. A literature search was performed in Pubmed http://www.pubmed.com using the words "nasopharyngeal carcinoma" and "radiosurgery". Only published papers using radiosurgery for treatment of persistent and recurrent nasopharyngeal carcinoma were considered, and papers on the use of radiosurgery as a boost treatment following primary radiotherapy were excluded. Papers containing sufficient information on patient and disease characteristics for calculation of scoring system were then included in this study. Prognostic scoring system of individual patient was calculated and actuarial tumor control and/or survival rates in different prognostic groups were then estimated and compared with our published results. The performance of the scoring system was also further examined comparing the area under the receiver operating characteristic (ROC) curve in the current and original patient groups.

## Results

A search in Pubmed using the specified keywords yielded 34 publications. Of these, 8 were reported by us and these were excluded from the validation study. The remaining 26 papers were carefully reviewed for inclusion into the study, and only 3 papers contained sufficient data on individual patient and disease characteristics for the purpose of estimation of prognostic scores. The prognostic score of individual patient was calculated based on the available information in the published manuscripts (Table 1). In case the prognostic score could not be calculated due to missing information, the mean score was obtained by estimating the lowest and highest score for that patient. The first paper by Chen et al [15] included 11 patients with rT4 disease with radiosurgery given to tumor extended to skull base and intracranium. In Chen's series, radiosurgery was performed using a modified linear accelerator, and a median dose of 14 Gy (range: 10 to 15 Gy) was delivered to the target. The calculated prognostic scores in Chen's series ranged from 0.32 to 0.8 with 10 patients classified as poor prognostic group and 1 patient as intermediate prognostic group. In the second paper by O'Donnell et al [16], two patients received radiosurgery using Gamma Knife (Elekta, Sweden) with a dose of 14 Gy, and both were classified as intermediate prognostic group. In the third paper by Kocher et al [17], 5 patients received radiosurgery using modified linear accelerator with a median dose of 20 Gy (range: 15 to 24 Gy) for local failure of nasopharyngeal carcinoma, and the calculated scores ranged from 0.75 to 1.21 thus all 5 patients were classified as poor prognostic group.

**Table 1:** Prognostic factors and calculated score of patients included in the current validation study

Patient	Age/score	rT stage/score	Persistent vs recurrent disease/score	Tumor volume (cc)/score	Prior salvage/score	Prognostic score	Prognostic group
1	53/1	rT4/1	Recurrent/1	0.91/0	NR	0.74	Poor
2	35/0	rT4/1	Recurrent/1	0.66/0	NR	0.52	poor
3	44/0	rT4/1	Recurrent/1	5.77/0	Yes/1	0.71	Poor
4	44/0	rT4/1	Recurrent/1	13.81/1	NR	0.8	Poor
5	55/1	rT4/1	Recurrent/1	1.31/0	NR	0.74	Poor
6	39/0	rT4/1	Recurrent/1	8.51/0	No/0	0.32	Intermediate
7	44/0	rT4/1	Recurrent/1	7.91/0	NR	0.52	poor
8	70/1	rT4/1	Recurrent/1	2.85/0	NR	0.74	Poor
9	63/1	rT4/1	Recurrent/1	15.77/1	NR	0.74	Poor
10	47/1	rT4/1	Recurrent/1	6.43/0	NR	0.74	Poor
11	38/0	rT4/1	Recurrent/1	13.03/1	NR	0.8	Poor
12	51/1	rT4/0	Recurrent/1	3.3/0	No/0	0.49	Intermediate
13	69/1	rT4/0	Recurrent/1	<10/0	No/0	0.49	Intermediate
14	58/1	rT4/1	Recurrent/1	8/0	Yes/1	0.93	Poor
15	47/1	rT4/1	NR	33/1	No/0	0.75	Poor
16	60/1	rT4/1	NR	60/1	No/0	0.75	Poor
17	61/1	rT4/1	Recurrent/1	22/1	Yes/1	1.21	Poor
18	51/1	rT4/1	Recurrent/1	52/1	Yes/1	1.21	Poor

NR: Not reported

**Figure 1:**
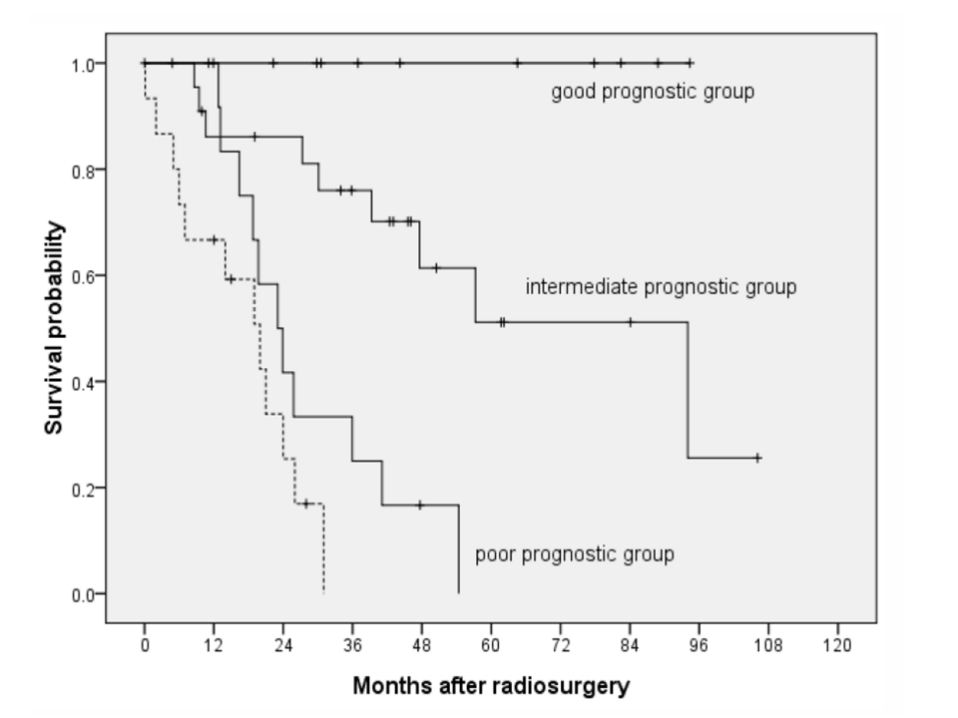
**Comparison of survival curves according to prognostic groups from original patient group (solid line) and the current patient group (dashed line)**. For current patient group, only the poor prognostic group was shown due to absence or small number of patients in other prognostic groups.

**Figure 2:**
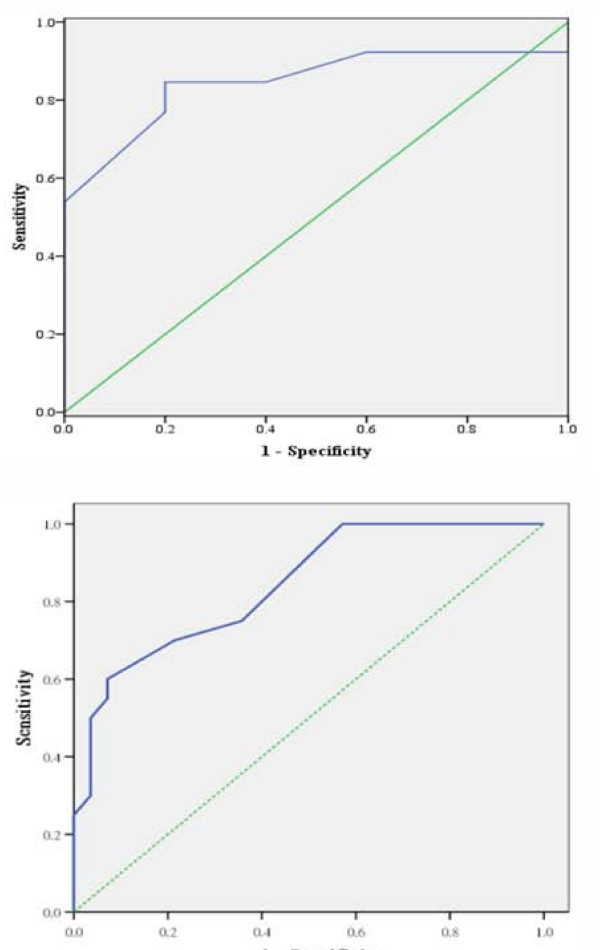
**Receiver operating characteristic curve for predicting overall survival after radiosurgery using prognostic scoring system in current patient group (top) and original patient group (bottom)**.

Data of all these patients were pooled together according to their prognostic group for comparison. Only overall survival rate was analyzed since local control status was not clearly defined as an endpoint in most patients. In the current cohort, the actuarial 3-year survival rates in the intermediate and poor prognostic groups were 67% and 0%, respectively. These results were comparable to the observed 3-year survival rate of 74% in the intermediate prognostic group and 31% in the poor prognostic group as described in our original report. Figure 1 shows the survival curves according to prognostic groups in the current and original cohorts. Although none of the patients in the current cohort belonged to the good prognostic group and there were few in the intermediate prognostic group, the survival curve of those assigned to the poor prognostic group was similar in both cohorts, suggesting that the scoring system is at least useful in predicting poor outcome in poor prognostic group after radiosurgery. To further assess the performance of the prognostic scoring system, the area under the ROC curve was calculated. If the performance of the scoring system resembles flipping a coin in predicting treatment outcome, the area under the ROC curve is close to 0.5. If the area is 1.0, then the model has 100% sensitivity and specificity in predicting outcome regardless of cut-off point. Figure 2 shows the ROC curves for overall survival for the current cohort and the original cohort used to design the scoring system. The calculated area under the ROC curve for the current cohort was 0.846 (95% C.I.: 0.66, 1.03) which is comparable to 0.841 (95% C.I.: 0.731, 0.952) in the original cohort, suggesting good predictive performance using the scoring system.

## Discussion

Treatment of local recurrence of NPC is usually recommended since a proportion of patients with local failure can still be successfully salvaged. In a retrospective review of 319 patients with isolated local failure of NPC, patients who received salvage treatment had a significantly better survival rate than those without salvage treatment, and patient who received surgery or re-irradiation had a better survival rate than those treated by chemotherapy alone [18]. Several salvage treatments including nasopharyngectomy, brachytherapy and external beam re-irradiation are commonly employed for local failure of NPC, and the choice of treatment is largely determined by the extent and site of recurrence as well as available expertise. For patient with recurrent disease that is amenable to more than one treatment option, few reports are available to address the relative efficacy of different types of salvage treatment [19], and the decision is largely a matter of patient and/or clinician preference. Since prospective randomized trial to compare different treatments in salvaging local failure is difficult to conduct due to the falling incidence of recurrence and the highly specialized nature of different salvage treatments, it is important to review prognostic factor in order to identify patients that are likely to benefit from specific type of salvage treatment. Our previously published prognostic scoring system is a simple and objective way to assess possible benefits of radiosurgery for local failures of NPC.

The prognostic factors included in the scoring system have also been identified in patients with recurrent NPC treated by surgery, brachytherapy and/or external beam re-irradiation. Most series also reported T stage and time to recurrence as significant prognostic factors for local control and/or survival [6,7,20,21]. The most consistent prognostic factor being reported was rT stage, and patients treated for advanced T stage generally suffered from poor tumor control and short survival time. Despite these similarities in prognostic factors, our scoring system cannot be applied directly to patients using salvage treatments other than radiosurgery, since the system is optimally modeled to the original data based on a group of patients with disease that were technically feasible for radiosurgery. Thus the weighting for rT4 disease is relatively small in the scoring system since majority of our patients did not have rT4 disease. In clinical practice, rT4 is an important poor prognostic factor and likely to carry a heavier weighting in patients treated by other means.

There are two major limitations of the current patient data set that we used to validate our scoring system. One limitation is the relatively small number of patients due to the lack of individual patient data available from most published reports. The other limitation is over-representation of patients in the poor prognostic group, reflecting the selection of those with relatively poor prognosis for radiosurgery in the literature, thereby limiting the validation of the scoring system to this particular prognostic group only. We are currently applying the prognostic scoring system in our institution to select patients with local failures of NPC for radiosurgery. This prospective exercise will provide additional data useful for validation in patients with good and intermediate prognostic groups since these are the patients likely to be selected for radiosurgery.

## Conclusion

The previously published prognostic scoring system demonstrated good prediction of treatment outcome after radiosurgery in a small group of NPC patients with poor prognosis. Prospective study to validate the scoring system is currently being carried out in our institution.

## Competing interests

The authors declare that they have no competing interests.

## Authors' contributions

DTTC designed the scoring system, carried out the review of literature for validation, and drafted the masnucsript. KNH and SN participated in the study design and performed the statistical analysis. VL and JT participated in the design of the scoring system and helped to draft the manuscript. All authors read and approved the final manuscript.

## Pre-publication history

The pre-publication history for this paper can be accessed here:

http://www.biomedcentral.com/1471-2407/9/131/prepub
